# Endoscopic full-thickness resection of a rectal submucosal tumor with the double-tunnel bridge formation method: a case report

**DOI:** 10.1055/a-2771-4546

**Published:** 2026-03-05

**Authors:** Naoya Toyoshima, Masau Sekiguchi, Hiroyuki Takamaru, Yukihide Kanemitsu, Susumu Hijioka, Toshihiro Haga, Yutaka Saito

**Affiliations:** 1 68380 Endoscopy Division, National Cancer Center Hospital, Tokyo, Japan; 2 13874 Department of Colorectal Surgery, National Cancer Center Hospital, Tokyo, Japan; 3 13874 Department of Hepatobiliary and Pancreatic Oncology, National Cancer Center Hospital, Tokyo, Japan; 4 13874 Department of Pathology, National Cancer Center Hospital, Tokyo, Japan

Endoscopic submucosal dissection (ESD) is a treatment option for colorectal epithelial lesions that enables en bloc resection of large or fibrotic lesions 1 . In Western countries, endoscopic full-thickness resection (EFTR) is often selected for difficult lesions, particularly those with severe fibrosis or non-lifting characteristics 2 3 . In Japan, where ESD originated, several technical methods – such as the double-tunnel method and the bridge formation method (BFM) – have been developed to facilitate resection in challenging cases 4 . In addition, peranal endoscopic myectomy (PAEM) has recently emerged as a minimally invasive option for lesions involving the muscularis propria, highlighting the usefulness of double-tunnel creation for controlled myectomy 5 . Building on these advancements, we applied double-tunnel formation and BFM to EFTR to achieve precise dissection and safe full-thickness resection of deeply invasive submucosal tumors (SMTs).

**Fig. 1:**
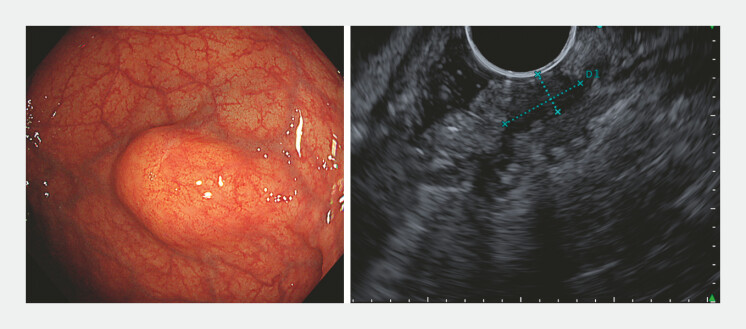
A 15-mm submucosal tumor located in the lower rectum. Endoscopic ultrasonography showing an 11-mm extramural hypoechoic lesion.

**Fig. 2:**
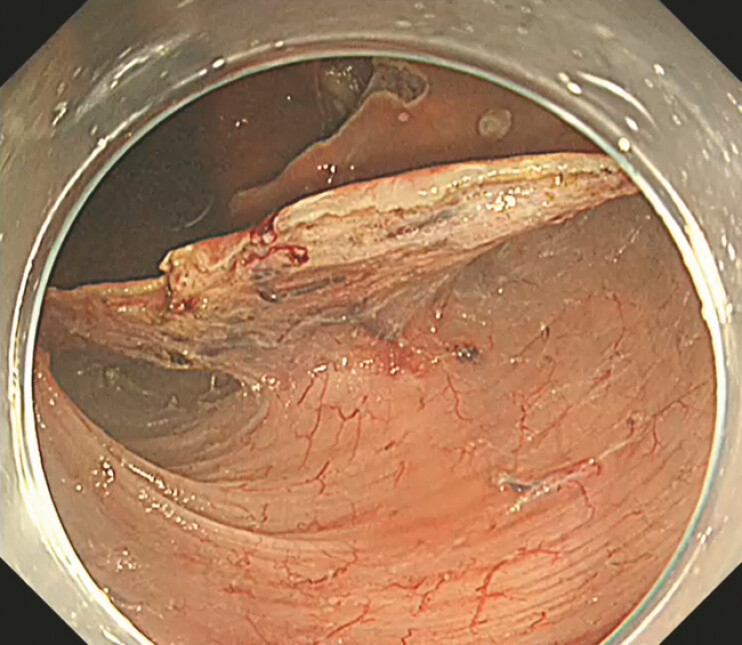
Wide mucosal incision and creation of two submucosal tunnels using the bridge formation method (BFM).

**Fig. 3:**
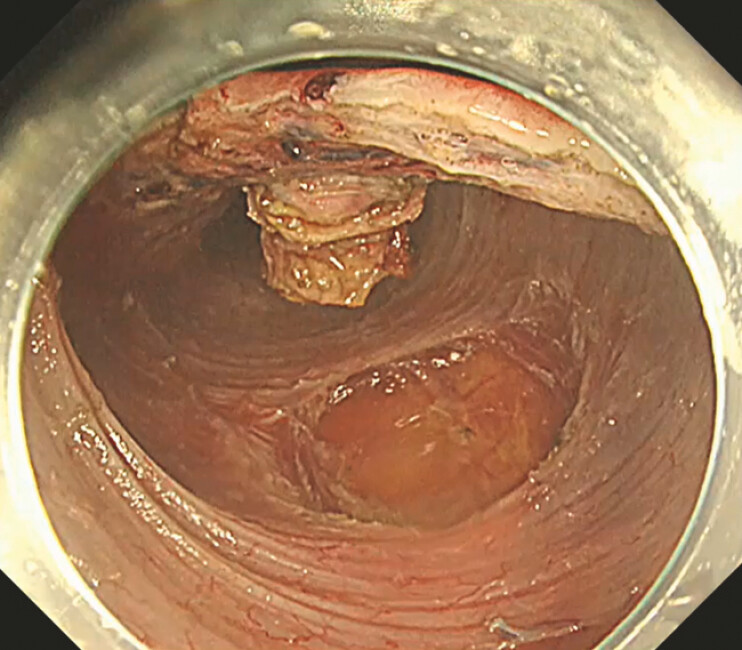
Full-thickness resection performed only at the area containing the tumor by utilizing natural traction.

**Fig. 4:**
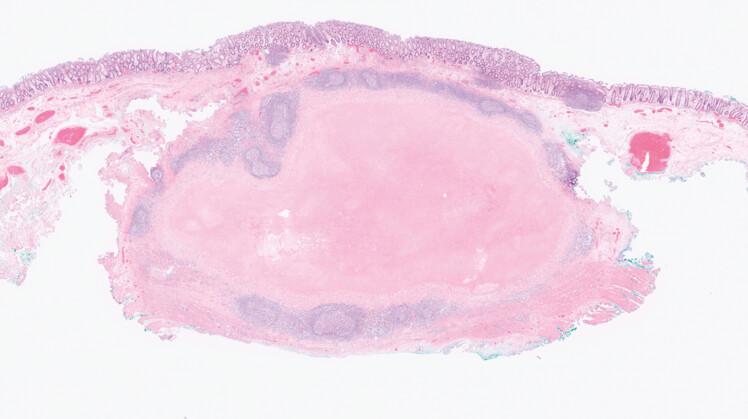
Granulomatous inflammation observed in the SMT-like lesion. SMT, submucosal tumor.

**Fig. 5:**
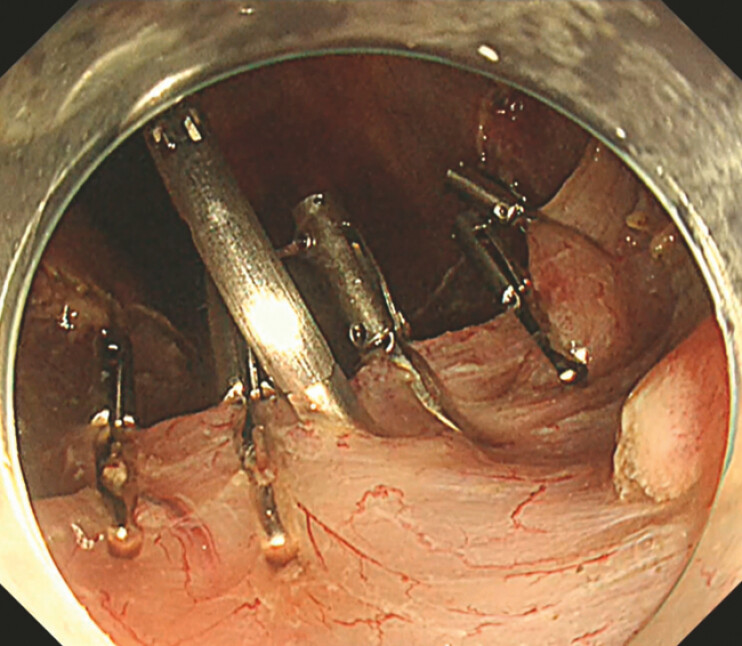
Closure of the full-thickness defect using clips.

A 74-year-old woman was referred after a positive fecal immunochemical test. Colonoscopy revealed a rectal SMT, and both boring biopsy and endoscopic ultrasound (EUS)-guided fine-needle aspiration were non-diagnostic. EUS showed an 11-mm hypoechoic submucosal mass partially extending into the muscularis propria ( Fig. 1 ). Endoscopic resection was performed for diagnosis ( Video 1 ). Two submucosal tunnels were created, but the lesion was not visualized, suggesting deeper involvement ( Fig. 2 ). Inner circular muscle resection was initiated, and with the BFM approach, the overlying mucosa was preserved to provide natural traction and clarify the dissection plane 2 . The lesion penetrated the muscularis propria and was removed en bloc as a full-thickness specimen ( Fig. 3 , Fig. 4 ). The defect was closed with a Mantis clip and additional standard clips, achieving secure ( Fig. 5 ) closure. The patient recovered uneventfully.

DBFR-assisted endoscopic full-thickness resection of a rectal submucosal tumor, showing double-tunnel creation, natural traction using the mucosal bridge, selective full-thickness dissection, and clip closure of the defect. DBFR, double-tunnel bridge formation.Video 1

This novel technique, termed the double-tunnel bridge formation method-assisted EFTR (DBFR), offers three major advantages: (i) the double-tunnel approach limits the extent of muscle resection; (ii) BFM provides natural traction and a clear dissection plane without additional devices; and (iii) the limited full-thickness defect permits secure closure with clips.

Endoscopy_UCTN_Code_TTT_1AQ_2AD_3AD
